# Calendar

**DOI:** 10.1155/S1463924684000225

**Published:** 1984


					Journal of Automatic Chemistry, Volume 6, Number 2 (April-June 1984), page 97
Calendar
Editor's note
Organizers of conferences, seminars, etc.
should send details for inclusion in the
calendar as soon as the relevant infor-
mation is available and not later than three
months before the event.
Course on Testing Thermal Images
To be held from 16 to 18 October 1984 in
Kent, UK.
Further informationfrom Sira Ltd, South
Hill, Chislehurst, Kent BR7 5EH, UK.
The Analysis of Solid Samples by Atomic
Emission and X-ray Fluorescence
Spectrometry
To be held on 17 October at Tioxide UK
Ltd, Stockton-on-Tees, UK.
Further informationfrom Miss P. E. Hut-
chinson, Analytical Division, Royal
Society of Chemistry, Burlington House,
London W1 VOBN.
August 1984
9th Conference of the Australian and New
Zealand Society for Mass Spectrometry
To be held from 27 to 31 August 1984 at
the Australian National University,
Canberra, Australia.
Further informationfrom Dr M. J. Lacey,
Secretary, ANZSMS Conference, CSIRO
Division of Entomology, GPO Box 1700,
Canberra, ACT 2601, Australia.
September 1984
Analyticon 84: The Second Conference for
Analytical Science organized by the
Scientific Manufacturers' Association of
Great Britain in Association with The
Royal Society of Chemistry and The
Chromatography Society
To be held from 4 to 6 September at The
Barbi6an Centre, London.
Further informationfrom Mr G. C. Young,
SIMA, Leicester House, 8 Leicester
Street, London WC2H 7BN.
Third European Symposium on Thermal
Analysis and Calorimetry
To be held from 9 to 15 September '1984 at
the Congress-Center-Casino Interlaken
in Interlaken, Switzerland.
Further informationfrom ESTAC 3 84, Dr
E. Marti. Im Langen Lob 181, CH 4054
Basel, Switzerland.
October 1984
2nd International Congress on
Automation and New Technology in the
Clinical Laboratory
To be held from 15 to 18 October 1984 in
Barcelona, Spain.
For further information contact 2nd
International Congress on Automation and
New Technology in the Clinical Labor-
atory, IV Confreso Nacional de la
Sociedad Espafiola de Quimica Clinica,
Apartado de Correos 543, Barcelona,
Spain.
Second International Harmonization
Symposium
To be held from 25-27 October 1984 in
Washington, DC.
Further information from Mrs K.
Fominaya, Association ofOfficial Analyti-
cal Chemists, Suite 210, 1111 N. 19th
Street, Arlington, Virginia 22209, USA.
Developments in Atomic Spectroscopy
To be held on 31 October 1984 at the
University of Birmingham, UK.
Further information from Miss P. E.
Hutchinson, Analytical Division, Royal
Society of Chemistry, Burlington House,
London W1 VOBN.
November 1984
23rd Annual Eastern Analytical
Symposium
To be held from 13 to 16 November 1984
at the New York Penta Hotel, New York,
USA.
Further information from Dr S. D. Klein,
EAS Publicity, Merck & Co., Inc.,
PO Box 200/RSOL-106, Rahway, New
Jersey 07065, USA.
Course on Microprocessor Familiariz-
ation
To be held from 19 to 20 November 1984
in Kent, UK.
Further information from Sira Ltd (see
above).
14th Annual Symposium on the Analytical
Chemistry of Pollutants and the 3rd
International Congress on Analytical
Techniques in Environmental Chemistry
To be held from 22-24 November 1984 at
the Palacio de Congresos in Barcelona,
Spain.
P urther information from 14th
Annual Symposium--3rd International
Congress--Expoquimia, Avda. Reina M.
Christina, Barcelona 4, Spain.
1985 MEETINGS
36th Pittsburgh Conference and
Exposition on Analytical Chemistry and
Applied Spectroscopy
To be held from 25 February to
March 1985 at the Convention Center,
New Orleans, Louisiana, USA.
Further information from David R. Weill
III, Shady Side Academy, 423 Fox Chapel
Road, Pittsburgh, Pennsylvania 15238,
USA.
Product Design Assurance in Engineering
To be held from 11 to 13 June 1985 at the
Wembley Conference Centre, London.
Further informationfrom Mrs H. M. W.
Gibbons, Society of Environmental
Engineers, Owles Hall, Buntingford,
Hertfordshire, UK.
IUPAC 1985
To be held from 9 to 13 September 1985 in
Manchester, UK.
Further information from The Royal
Society of Chemistry, Burlington House,
London W1 V OBN.
Colloquium Spectroscopium Internation-
ale XXIV
To be held from 15 to 21 September
1985 in Garmisch-Partenkirchen, FR
Germany.
Further information from CSI XXIE
Organisationsbfiro, Institut ffir Spektro-
chemic und angewandte Spektroskopie,
Postfach 778, D4600 Dortmund 1, FR
Germany.
1986 MEETING
SAC 86/3rd BNASS: An International
Conference on Analytical Chemistry
To be held from 20-26 July 1986 at the
University of Bristol, UK.
Purther information from The Secretary,
Analytical Division, Royal Society of
Chemistry, Burlington House, London
W1 VOBN.
97

				

